# Exploring the Feasibility of Estrogen Replacement Therapy as a Treatment for Perimenopausal Depression: A Comprehensive Literature Review

**DOI:** 10.3390/medicina60071076

**Published:** 2024-06-30

**Authors:** Xiuting Xiang, Praneetha Palasuberniam, Rahmawati Pare

**Affiliations:** Department of Biomedical Science, Faculty of Medicine and Health Sciences, Universiti Malaysia Sabah, Kota Kinabalu 88400, Malaysiapraneetha@ums.edu.my (P.P.)

**Keywords:** perimenopausal depression, estrogen replacement therapy, treatment

## Abstract

Perimenopausal depression (PMD) is a psychological disorder that occurs in women during perimenopause. In addition to the common clinical symptoms of depression, it often manifests as a perimenopausal complication, and its notable cause is the decline in estrogen levels. Despite numerous studies and trials confirming the benefits of estrogen replacement therapy (ERT) for PMD, ERT remains unapproved for treating PMD. Therefore, we conducted a literature search using selected keywords in PubMed and Google Scholar to write a review discussing the feasibility of using ERT for PMD. This review examines the potential of ERT for PMD in terms of its underlying mechanisms, efficacy, safety, and time window. These four aspects suggest that ERT is a viable option for PMD treatment. However, the risk of thrombosis and stroke with ERT is a matter of contention among medical experts, with a paucity of clinical data. Consequently, further clinical trial data are required to ascertain the safety of ERT.

## 1. Introduction

Depression is a prevalent and serious mental health condition that affects individuals globally, with women being disproportionately affected in comparison to men [1]. Specifically, during the perimenopausal phase, which typically occurs between ages 42 and 52, women may experience perimenopausal depression (PMD) as a subtype of major depressive disorder [2]. A recent random effects model suggests that the pooled prevalence of depression was 35.6% in menopausal women, 33.9% in perimenopausal women, and 34.9% in postmenopausal women [3]. Generally, depression is characterized by persistent feelings of sadness and a diminished interest in activities, which can significantly impair relationships, productivity, and overall quality of life [4]. However, in addition to depression itself, PMD is accompanied by a variety of symptoms of perimenopause syndrome, such as insomnia, mood swings, and weight changes [5], and it also increases the risk of developing diseases such as osteoporosis and cardiovascular diseases [6]. Although declining estrogen levels are the primary cause of these symptoms, the presence of these symptoms exacerbates the manifestations of PMD [7]. Therefore, PMD is not merely the result of estrogen deficiency; rather, it is also a complex condition triggered by multiple perimenopausal symptoms.

Currently, there are no pharmacologic treatments specifically designed to target PMD. The general approach is to continue with the initial depression treatment plan. Antidepressants remain the first-line treatment for PMD [8]. Nevertheless, antidepressants have demonstrated efficacy in alleviating the majority of depressive symptoms in PMD patients. However, they were associated with a range of adverse effects, including nausea, dizziness, weight changes, addiction, and withdrawal symptoms [9]. Moreover, antidepressants were ineffective in alleviating the symptoms of vasoconstriction [10], osteoporosis [11,12], coronary artery atherosclerosis [13,14], insomnia [15], genitourinary syn-drome of menopause [16], and skin laxity [17] in perimenopausal women due to declining estrogen. One method for developing a cure for a disease is to identify the pathogenesis and then treat it according to the underlying cause.

The etiology and pathophysiology of PMD remain elusive, with potential associations with endocrine hormones as well as genetic and educational factors [18]. Nevertheless, women with PMD exhibit a distinctive profile compared to other depressed women, characterized by a decline in estrogen levels and menopausal symptoms. This review article examines the potential efficacy of estrogen replacement therapy (ERT) for the treatment of perimenopausal depression (PMD), with a particular focus on its feasibility.

## 2. Materials and Methods

In this comprehensive review, we compiled and examined the literature on estrogen replacement therapy for perimenopausal depression based on keyword searches. The objective of this study was to explore the feasibility of using estrogen replacement therapy in women with perimenopausal depression. In particular, the mechanisms, efficacy, safety, and optimal timing of application were investigated.

### 2.1. Search Strategy

**Figure 1:**
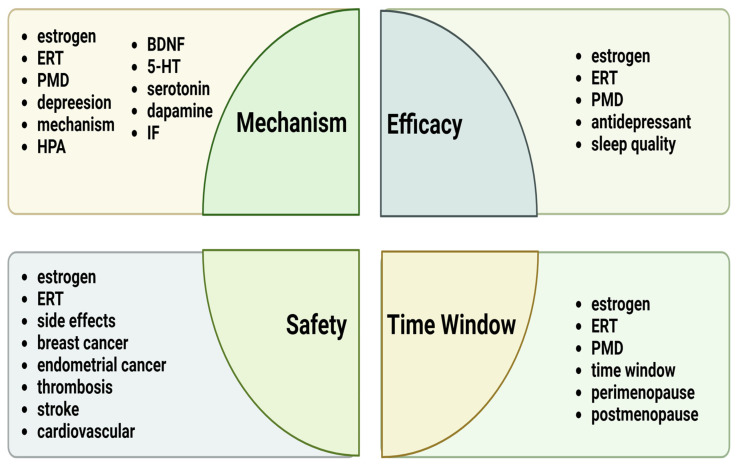
Summary of the main keywords discussed in the four sections of this review. Abbreviations: ERT, estrogen replacement therapy; PMD, perimenopausal depression; HPA, hypothalamic–pituitary–adrenal; IF, inflammatory factor.

A literature search was conducted using selected keywords in the following databases: PubMed and Google Scholar. The keywords selected for this study included the following search terms: “estrogen replacement therapy”, “estrogen”, “perimenopausal depression”, “HPA”, “inflammatory factor”, “BDNF”, “dopamine”, “serotonin”, “5-HT”, “sleep quality”, “antidepressant”, “side effects”, “breast cancer”, “endometrial cancer”, “thrombosis”, “stroke”, “time window”, “cardiovascular”, “perimenopause”, and “postmenopause”. These terms are specifically detailed in the four sections as shown in Figure 1.

### 2.2. Selection Criteria

The literature was limited to English, with publication dates of 2000 or later. Eligible studies were filtered based on the search results. The results were grouped based on the topics discussed after screening the abstracts. We also obtained the full text of the articles and screened the references to identify additional articles not included in the original search. In the case of clinical trials, only studies that reported explicit methods, patient age, and clear conclusions were included.

## 3. Results and Discussion

This review included *n* = 63 articles, of which *n* = 18 articles introduced the topic, and *n* = 14 addressed the possible mechanisms by which estrogen replacement therapy acts on perimenopausal depression through hypothalamic–pituitary–adrenal (HPA) effects. Subsequently, *n* = 9 articles described the effects of ERT on PMD, including summarizing the efficacy of estrogen-only therapy in PMD (4), the relationship between sleep quality and depression (1), the relationship between estrogen and antidepressants (2), and the efficacy of estrogen combined with antidepressants in PMD (2). In addition, *n* = 14 articles summarized the effects of common treatment complications associated with ERT, and *n* = 7 articles described the optimal treatment window for ERT, including definitions and explanations of the time-treatment window (3), the effects of exceeding the ERT treatment window on depression (2), and the effects of ERT performed during the treatment window on other diseases (2). Finally, in the conclusion section, *n* = 1 article described the trend in PMD incidence.

### 3.1. ERT for PMD: Feasibility in Mechanism

Despite the identification of numerous mechanisms as potential contributors to the PMD process, ovarian dysfunction appears to be a pivotal factor that links these various elements [19]. Hormones in the ovaries mainly include estrogen and progesterone, and PMD is most strongly associated with the former [20]. Estrogen originates from the ad-renal glands in addition to the ovaries, so estrogen is primarily regulated by the adrenal glands as a woman enters perimenopause, which brings us to the hypothalamic–pituitary–adrenal (HPA axis). Researchers believe that inflammation and hyperactivity of the HPA axis significantly contribute to the etiology of depression [21].

In the absence of pathological conditions, the HPA axis functions in a regulated and balanced manner. The process typically involves the following steps: The release of corticotropin-releasing hormone (CRH) from the hypothalamus initiates the release of adrenocorticotropic hormone (ACTH) from the lower pituitary gland, which in turn stimulates the adrenal glands, resulting in a sustained increase in cortisol (including mineralocorticoids, glucocorticoids, and sex hormones) in the body. When cortisol is overproduced, it acts on the hypothalamus and pituitary gland, resulting in a negative feedback effect [22].

**Figure 2:**
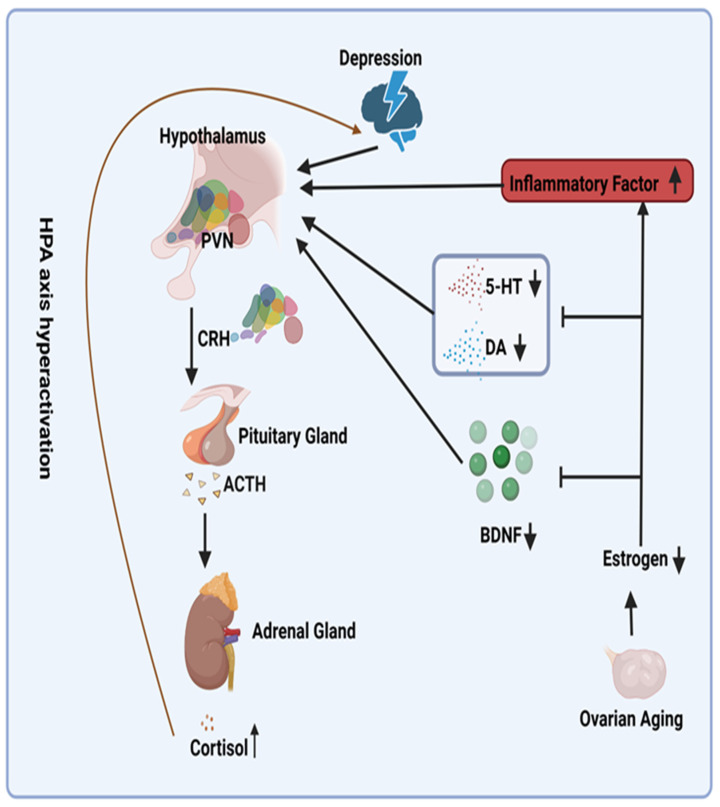
Potential Mechanisms for Overactivation of the Hypothalamic–Pituitary–Adrenal Axis Due to Declining Estrogen Levels. Abbreviations: PVN, paraventricular nucleus; ACTH, adrenocorticotropin-releasing hormone; CRH, cortisol hormone; 5-HT, serotonin; DA, dopamine; BDNF, brain-derived neurotrophic factor. Symbols: ↑, increase; ↓, decrease; I, inhibit.

Patients with depression frequently demonstrate hyperactivity of the HPA axis due to impaired glucocorticoid-mediated negative feed-back mechanisms [23,24]. The decline in estrogen levels that occurs with ovarian aging serves to exacerbate this hyperactivity (Figure 1). From a mechanistic perspective, reduced estrogen levels result in decreased levels of serotonin (5-HT) and dopamine (DA), two crucial neurotransmitters for mood regulation [25]. This further stimulates the HPA axis [26,27]. Furthermore, reduced estrogen levels induce a pro-inflammatory state in menopausal women [28]. The inflammatory cytokines interleukin-1 (IL-1), interleukin-6 (IL-6), and tumor necrosis factor alpha (TNFα), which have been demonstrated to be linked to this state, have been shown to activate the HPA axis [29]. In addition, reduced estrogen levels are associated with diminished levels of brain-derived neurotrophic factor (BDNF) [30]. A significant decrease (60%) in BDNF has been demonstrated to impact the basal activity of the hypothalamic–pituitary–adrenal (HPA) axis [31]. Consequently, it is postulated that reduced estrogen levels result in decreased BDNF levels, thereby activating the HPA axis. Conversely, research has indicated that ERT can mitigate the HPA axis response to emotional stress in postmenopausal women [32], potentially providing a mechanistic explanation for addressing this issue (Figure 2).

Consequently, estrogen replacement therapy appears to be a viable option for theoretically addressing the underlying pathogenesis of PMD. 

### 3.2. ERT for PMD: Feasibility in Effectiveness

#### 3.2.1. Efficacy of Estrogen-Only Therapy in PMD

To assess the effectiveness of ERT in the treatment of PMD, a comprehensive literature search for clinical trials on estrogen and perimenopausal depression was conducted. The results revealed a noticeable decline in the number of trials conducted after 2005, with most on this topic taking place between 2000 and 2005. Therefore, we included trials from 2000 and beyond in the review (Table 1).

Of the four clinical trials listed in the table, three demonstrated that estrogen therapy significantly improved perimenopausal depression. Only one of the trials did not demonstrate a significant difference. Notably, although this clinical trial indicated that estrogen therapy has no significant therapeutic effect on PMD compared to placebo, the study also showed that estrogen therapy can significantly improve sleep quality in PMD patients [35]. In contrast, sleep problems are a common clinical manifestation in patients with PMD, and they can further affect the symptoms of PMD [37]. This finding suggests that estrogen therapy may have beneficial effects on PMD by improving sleep quality, despite its limited direct therapeutic impact compared to a placebo. Therefore, it is reasonable to conclude from previous clinical trials that during perimenopause, the administration of transdermal estrogen at a dosage of 0.1 mg per day for a period of 4 weeks or 12 weeks and oral estrogen at a dosage of 1.25 mg per day for a period of 4 weeks may have the potential to benefit patients with PMD.

Consequently, in terms of its pharmacological effects, estrogen therapy only is a viable treatment option for PMD.

#### 3.2.2. Efficacy of Estrogen Combined with Antidepressants in PMD

We previously discussed the effectiveness of estrogen alone in treating PMD. However, it has been reported that estrogen may enhance the efficacy of antidepressant medications in the treatment of depression [38,39]. Therefore, we also searched for clinical trials of estrogen plus antidepressants for perimenopausal depression after 2000 but found only two relevant trials (Table 2). Despite the limited number of trials, these two trials were consistent in their conclusions that estrogen combined with antidepressant therapy had a greater effect in alleviating PMD than either estrogen or antidepressant alone.

It is regrettable that there is a paucity of clinical trial data on this treatment strategy. Future studies should endeavor to increase the sample size and allow for long-term follow-up studies to enhance the statistical power of the studies and the generalizability of the results. In conclusion, despite the limited research on the combination of estrogen and antidepressants for the treatment of acromegaly, the preliminary results indicate potential therapeutic efficacy and provide motivation and a basis for future in-depth research and validation of this therapeutic strategy.

Overall, current clinical trials suggest that both estrogen alone and estrogen combined with antidepressants are effective regimens for PMD. However, the combined regimen appears to be more efficacious.

### 3.3. ERT for PMD: Feasibility in Safety

Safety is one of the key factors in determining the feasibility of a drug. Therefore, given the therapeutic effect of estrogen replacement therapy on PMD, we sought to evaluate the safety of this treatment. The most prevalent complications associated with estrogen replacement therapy include endometrial cancer, breast cancer, thrombosis, and cardiovascular disease [42]. Given the dearth of empirical evidence regarding the complications of estrogen replacement therapy for major diseases, a comprehensive review of clinical trials conducted from 2000 to the present was conducted. Table 3 presents the results of the studies in tabular form.

Based on a review of prior clinical trials (Table 3), there is currently no evidence to suggest that estrogen replacement therapy increases the risk of endometrial cancer or cardiovascular disease.

Regarding breast cancer, 10 clinical studies have explored the association between ERT and breast cancer risk. However, only one study indicated that oral ERT may increase breast density associated with breast cancer risk. This variation may be linked to the duration of estrogen therapy, the mode of administration, and the age at which estrogen therapy was initiated. Specifically, seven of these studies involved women aged 50 to 79 years receiving daily oral doses of 0.625 mg estrogen for more than 5.7 years, none of which demonstrated an increased risk of breast cancer with oral ERT. The sole study suggesting a potential increase in breast cancer risk with oral ERT utilized a similar treatment regimen but over a duration of only 1 to 2 years [49]. This suggests that the potential effectiveness of adjusting treatment duration in mitigating the risk of breast cancer may be associated with ERT. Another potential explanation for this discrepancy is that the sample size of this clinical trial was significantly reduced in comparison to the other groups, which may have introduced some degree of error because two other clinical trials, each also lasting 1 to 2 years—one involving low-dose (0.014 mg) transdermal estrogen therapy [50] and the other involving a relatively younger cohort (aged 45 to 60 years) [53]—demonstrated that ERT did not increase the risk of breast cancer. So together, factors such as the duration of estrogen therapy, mode of administration, dosage, and age of therapy initiation may be critical in assessing the relationship between ERT and breast cancer risk. These clinical trials may suggest that initiating estrogen supplementation relatively early and employing transdermal estrogen administration are important strategies to mitigate the risk of breast cancer associated with ERT. In conclusion, this evidence suggests that the complications of ERT leading to breast cancer might be effectively circumvented by modifying the dose, mode of administration, timing of administration, and age of initiation of treatment.

The impact of ERT on thrombosis and stroke risk is a significant finding in the current study. A review of just four relevant clinical studies indicated that two of them suggested that ERT might elevate the risk of thrombosis or stroke. Consequently, the impact of ERT on these risks varies considerably across different clinical contexts, presenting considerable complexity. The review of these four pertinent clinical studies yielded the following insights: Among American women who underwent hysterectomy, oral administration of 0.625 mg estrogen for 5.9 years did not increase the risk of thrombosis or stroke [54]. However, extending the treatment to 6.8 years appeared to heighten the risk [52]. Conversely, in Black African women, even with oral estrogen at 0.625 mg for 7.2 years, there was no observed increase in thrombosis or stroke risk [45]. These findings highlight the significant impact of estrogen therapy duration on thrombosis and stroke risk, which varies among different population groups. Moreover, a pivotal finding from a final clinical case study indicated that individuals with a history of ischemic stroke or transient ischemic attack may be at an elevated risk of thrombosis and stroke, even with a low daily dose of 1 mg oral estrogen over a treatment period of just 2.8 years [55]. This highlights the importance of patients with a history of cardiovascular disease or related risk factors carefully considering the potential risks associated with estrogen therapy. Therefore, the risk of thrombosis and stroke associated with ERT requires consideration of ethnic disparities, duration of therapy, and the patient’s cardiovascular risk profile. Given the limitations of current clinical studies in adequately assessing these risks, future research should aim to more fully explore the interactions between these factors and validate these findings with a larger number of clinical studies to provide more precise clinical guidance.

In conclusion, the currently available evidence does not support the hypothesis that ERT increases the risk of breast cancer, endometrial cancer, and cardiovascular disease. Nevertheless, further clinical data are required to ascertain the impact of ERT on the risk of thrombosis or stroke.

### 3.4. ERT for PMD: Time Window Feasibility

In recent years, a “window of opportunity” has been identified as a potential time for more effective initiation of hormone replacement therapy (HRT). It is hypothesized that the efficacy of HRT is contingent upon the timing of its commencement, with the benefits being constrained to those initiated in the vicinity of the onset of menopause. Consequently, late initiation of HRT is considered to be unhelpful or even harmful [56,57,58]. Indeed, there is evidence from clinical studies that ERT therapy is not beneficial when initiated post menopause in depressed women [59,60]. It is fortuitous that patients with PMD are in the optimal time window for treatment. Furthermore, ERT for PMD during this window of time can also have beneficial effects on osteoporosis [61], Alzheimer’s disease [62], and other comorbid syndromes in perimenopausal women.

Consequently, in terms of the time-to-treatment window, ERT for PMD is a viable option, as it has a beneficial impact on other comorbidities in perimenopausal women.

## 4. Conclusions

As the economy has expanded at a rapid pace, the social pressure faced by women has gradually intensified. In particular, the prevalence of PMD in women has been on the rise year by year [63]. This paper examines the potential of ERT for the treatment of PMD in terms of its underlying mechanisms, efficacy, safety, and time window. The preceding four aspects demonstrate that ERT is a viable option for PMD treatment. However, whether ERT causes thrombosis and stroke remains controversial among medical experts and may be closely related to the patient’s health status, estrogen dose, and regimen. More clinical data are needed to further investigate and refine these concerns. Future research should focus on developing ways to mitigate the side effects of ERT. Modifying the method, dose, and duration of estrogen administration can achieve this. Only when we fully understand this complex issue can we effectively utilize it.

## Figures and Tables

**Table 1:** Clinical Trial Literature on Estrogen-only for Perimenopausal Depression from 2000 to the Present.

References	Treatment Program (Estrogen Dosage)	Method of Administration	Course of Treatment	Number for Statistics	Age	Curative Effect
[33]	0.1 mg	TE	12 w	50	40–45	Positive. Mean MADRS and BKMI scores were lower in women treated with TE than in women treated with placebo.
[34]	0.1 mg	TE	4 w	9	42–57	Positive. Six women had a MADRS score < 10 and a CGI score ≤ 2.
[35]	0.1 mg	TE	8 w	62	40–60	Negative. No significant differences were observed in the Center for Epidemiologic Studies Depression Inventory, the BDI scores, or the HRSD in patients treated with TE compared to those who received placebo. However, it does improve sleep quality in women with PMD.
[36]	1.25 mg	Oral	4 w	20	42–57	Positive. The scores on the MADRS, BDI, and CGI scales demonstrated improvement in patients.

Abbreviations: W, week; TE, transdermal estrogen; MADRS, Montgomery–Åsberg Depression Rating Scale; BKMI, Blatt–Kupperman Menopausal Index; CGI, Clinical Global Impression; HRSD, Hamilton Rating Scale for Depression; BDI, Beck Depression Inventory.

**Table 2:** **The** Clinical Trial Literature on Estrogen Combined with Antidepressants for Perimenopausal Depression from 2000 to Present.

Ref.	Treatment Program	Course of Treatment	Number	Age	Curative Effect
[40]	Oral estrogen 0.625 mg + Antidepressants (individualized)	6 w	17	40–60	Patients receiving combined estrogen and antidepressant therapy had lower HAM-D scores than those receiving antidepressant therapy with placebo.
[41]	Transdermal 0.1–0.2 mg estrogen + oral 10–20 mg fluoxetine	8 w	5	51–57	Patients treated with combined estrogen and fluoxetine had a greater amount of change in HDRS and BDI scores relative to baseline compared with estrogen only or fluoxetine only.

Abbreviations: W, week; HAM-D, Hamilton Rating Scale for Depression; HDRS, Hamilton Depression Rating Scale scores; BDI, Beck Depression Inventory.

**Table 3:** The Clinical Trial Literature on the Effects of Estrogen Replacement Therapy on Major Diseases, 2000–Present.

Ref.	Treatment Program (Estrogen Dosage)	Number	Age	Population	Method	Course of Treatment	Increased Risk of Serious Diseases			
							EC	BC	DVT/Stroke	CVD
[43]	1 mg	67	51–70	Menopausal women with genitourinary syndromes	Vaginal Cream	32 weeks	N	/	/	/
[44]	300 μg	408	52.5 ± 3.9	Postmenopausal women with moderate-to-severe menopausal symptoms	Pulse	1 years	N	/	/	/
[45]	0.625 mg	1616	50–79	Hysterectomized Black women	Oral	7.2 years	/	N	N	N
[46]	0.625 mg	10,739	50–79	Hysterectomized women	Oral	7.2 years	/	N	/	/
[47]	0.625 mg	10,739	50–79	Hysterectomized postmenopausal women	Oral	6.8 years	/	N	/	/
[48]	0.625 mg	10,739	50–79	Hysterectomized postmenopausal women	Oral	7.1 years	/	N	/	/
[49]	0.625 mg	435	50–79	Hysterectomized postmenopausal women	Oral	1–2 years	/	Y	/	/
[50]	0.014 mg	417	66 ± 5	Postmenopausal women without hysterectomy or breast cancer history	TE	1–2 years	/	N	/	/
[51]	0.625 mg	10,739	50–79	Hysterectomized postmenopausal women	Oral	7.1 years	/	N	/	/
[52]	0.625 mg	10,739	50–79	Hysterectomized postmenopausal American women	Oral	6.8 years	/	N	Y	N
[53]	0.625 mg	168	45–60	Healthy postmenopausal women with hysterectomy within the past 15 years and no history of breast cancer	Oral	2 years	/	N	/	/
[54]	0.625 mg	10,739	50–79	Hysterectomized postmenopausal American women	Oral	5.9 years	/	N	N	N
[55]	1 mg	664	Mean-71	Postmenopausal women with recent stroke or transient ischemic attack	Oral	2.8 years	/	/	Y	/

Abbreviations: N, No; Y, Yes; /, no mention; TE, transdermal estrogen; EC, endometrial cancer; BC, breast cancer; DVT, deep vein thrombosis; CVD, cardiovascular disease.
